# Supplementary material for: Reduced Kidney Function and Relative Hypocalciuria—Observational, Cross-Sectional, Population-Based Data
Source: J Clin Med. 2020 Dec 21;9(12):4133. doi: 10.3390/jcm9124133 (PMC7767498; doi:10.3390/jcm9124133)
Supplement: Supplementary file 1 [file jcm-09-04133-s001.pdf]

Supplementary Materials

Table S1. Gubbio study dataset: comparison between examinees included in analyses (complete collection for overnight urine and for morning urine after overnight fast) and examinees excluded from analyses (with missing collection of morning urine after overnight fast).

	Examinees Included in Analyses with Complete Collection for Overnight Urine and for Morning Urine Collection Under Fasting Conditions	Examinees Excluded From Analyses due to Missing Morning Urine Collection Under Fasting Conditions	*p* for Contrast ^
Number of examinees	3508	1162	
% women	55.7%	52.3%	0.051
Age, years	50.3 ± 16.7	49.1 ± 20.9	0.067
Body mass index, kg/m^2	26.8 ± 4.3	26.4 ± 4.6	0.023
eGFR, mL/min x 1.73 m^2	87.3 ± 16.8	85.5 ± 19.4	0.003
Overnight urine Ca/creatinine ratio, mg/g	125 ± 77	123 ± 80	0.403

^ by chi-square analysis or ANOVA.

Table S2. Gubbio study dataset: descriptive statistics by eGFR stratum.

	≥90	89–75	74–60	59–45	44–30	29–15	*p* for trend ^
N	1585	1128	635	140	17	3	
% men	57.1%	40.0%	25.8%	22.1%	23.5%	0.0%	<0.001
Age, years	39.1	54.2	65.3	72.9	80.4	72.7	<0.001
Body mass index, kg/m^2	25.9	27.3	27.7	27.7	26.9	26.9	<0.001

^ by chi-square analysis or ANOVA.

Table S3. Moli-sani study dataset: descriptive statistics by eGFR stratum.

	eGFR, mL/min × 1.73 m^2						*p* for trend ^
	≥90	89–75	74–60	59–45	44–30	29–15	
N	334	343	195	66	13	4	
% men	58.4%	49.0%	45.1%	45.5%	38.5%	50.0%	0.002
Age, years	53.6	60.2	64.8	70.4	72.9	75.8	<0.001
Body mass index, kg/m^2	27.6	28.8	30.1	29.1	29.6	29.9	<0.001

^ by chi-square analysis or ANOVA.

Table S4. Gubbio study dataset and Moli-sani study dataset: additional models of multivariate linear regression of urine Ca/creatinine ratio over eGFR.

Type of Urine Sample	Covariates in Multivariable Model		Difference in Urine Ca/Creatinine Ratio Per 10 mL Difference in eGFR *(95%CI)*	*p*
overnight urine ^	Age, weight, ending time of last meal, habitual intake of milk or yogurt, Ca supplementation, and vitamin D supplementation	Men	14.2 (11/17)	<0.001
		Women	17.6 (15/21)	<0.001
morning fasting urine ^	Age, weight, ending time of last meal, duration of fast before morning fasting urine, habitual intake of milk or yogurt, Ca supplementation, and vitamin D supplementation	Men	9.6 (7/12)	<0.001
		Women	11.3 (9/14)	<0.001
first-void morning urine ^^	Age, weight, Ca supplementation, and vitamin D supplementation	Men	7.7 (4/12)	<0.001
		Women	9.1 (4/14)	<0.001

^ Gubbio study dataset; ^^ Moli-sani study dataset.

Table S5. Moli-sani study dataset: logistic regression of prevalence of CKD-MBD metabolic abnormalities over urine Ca/creatinine ratio in men and women with reduced kidney function.

Dependent Variable		OR for 25 mg/g Lower Urine Ca/Creatinine Ratio (95%CI)	*p*
Hyperphosphatemia	Men	0.92 (0.56/1.50)	0.739
	Women	1.14 (0.96/1.35)	0.129
High serum PTH	Men	2.03 (0.92/4.49)	0.079
	Women	1.11 (0.84/1.48)	0.456
Low serum 1,25$(OH)_2D$	Men	2.34 (1.22/4.49)	0.010
	Women	1.93 (1.27/2.93)	0.002
Hypocalcemia	Men	1.12 (0.68/1.87)	0.655
	Women	0.84 (0.54/1.30)	0.432

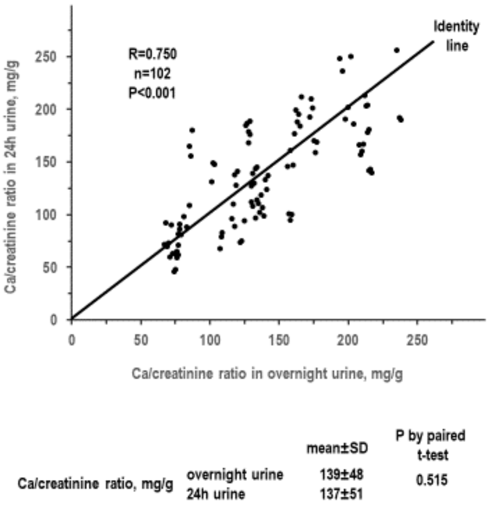

Figure S1. Gubbio study dataset, subgroup analyses: correlation and paired t-test for Ca/creatinine ratio between overnight urine and estimated same day 24 h urine.

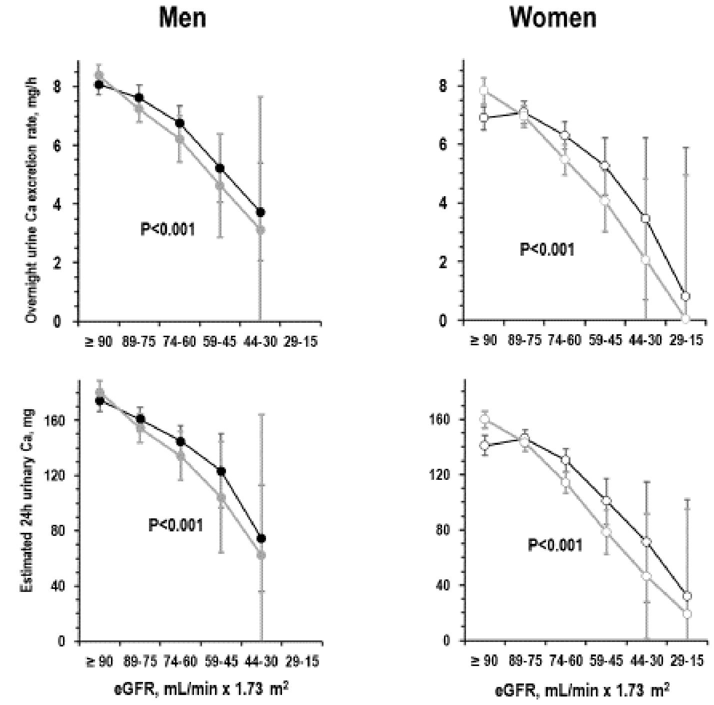

Figure S2. Gubbio study dataset: mean and 95%CI of overnight urine Ca excretion rate (upper panels) and of estimated 24 h urinary Ca (lower panels) by sex and eGFR stratum in uni-variate ANOVA and in ANOVA controlled for age, and weight (black line and grey line, respectively). *p* values are for trend in ANOVA. 24 h urinary Ca was estimated as Ca/creatinine ratio in overnight urine times estimated 24 h urinary creatinine. Number of individuals per eGFR stratum from left to right: men = 905, 451, 164, 31, 4, and 0; women = 680, 677, 471, 109, 13, and 3. No individual had eGFR < 15 mL/min × 1.73 m^2.

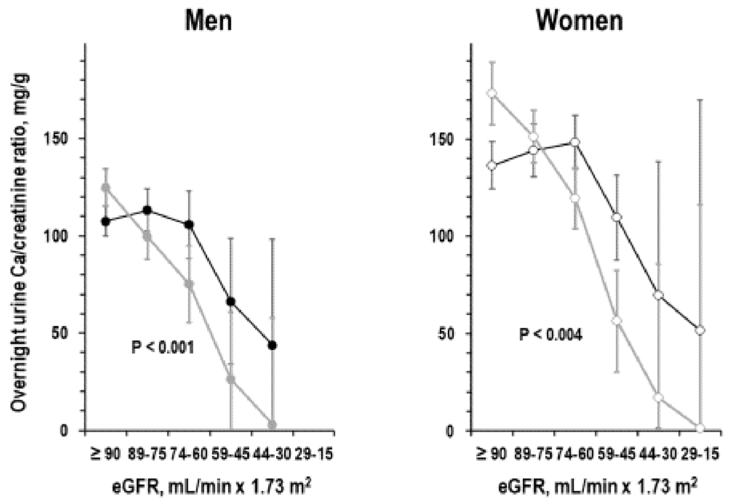

Figure S3. Gubbio study dataset: mean and 95%CI of Ca/creatinine ratio in overnight urine by sex and eGFR stratum in 1162 examinees excluded from main analyses for the lack of morning urine collection after overnight fast. Uni-variate ANOVA and ANOVA controlled for age, and weight (black line and grey line, respectively). P value is for trend in ANOVA. Number of individuals per eGFR stratum from left to right: men = 307, 159, 63, 17, 7, 0, and 1; women = 196, 166, 165, 69, 10, 2, and 0. No individual had eGFR < 15 mL/min × 1.73 m^2.

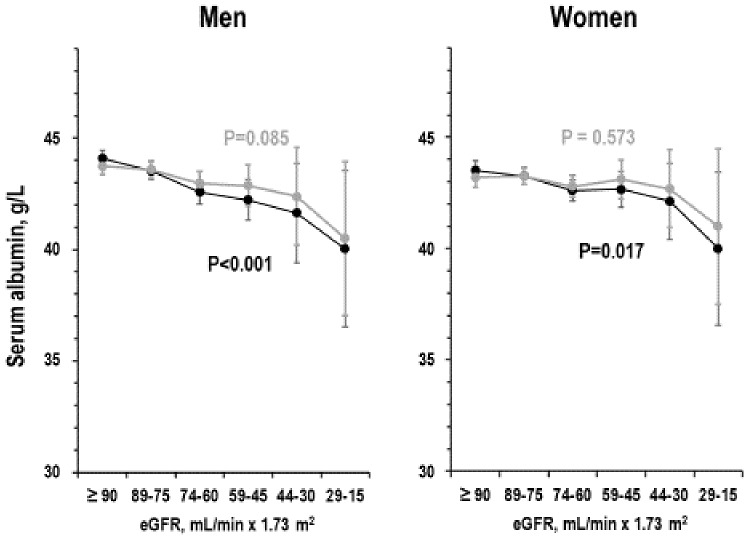

Figure S4. Moli-sani study dataset: mean and 95%CI of serum albumin by sex and eGFR stratum. P value is for trend in ANOVA. Number of individuals per eGFR stratum: men = 195, 168, 88, 30, 5, and 2; women = 139, 175, 107, 36, 8, and 2. No individual had eGFR < 15 mL/min × 1.73 m^2.
